# Cardiovascular MRI Feature-Tracking Strain Rate for Assessment of Diastolic Function

**DOI:** 10.1148/ryct.240447

**Published:** 2025-10-02

**Authors:** Jian L. Yeo, Abhishek Dattani, Aseel Alfuhied, Anna-Marie Marsh, Kelly S. Parke, Sarah L. Ayton, Lavanya Athithan, Joanna M. Bilak, Alastair J. Moss, Emer M. Brady, J. Ranjit Arnold, Prathap Kanagala, Christopher D. Steadman, Matthew P. M. Graham-Brown, Melanie J. Davies, Anvesha Singh, Iain B. Squire, Leong L. Ng, Gaurav S. Gulsin, Gerry P. McCann

**Affiliations:** ^1^Department of Cardiovascular Sciences, University of Leicester and the National Institute for Health and Care Research (NIHR) Leicester Biomedical Research Centre, Glenfield Hospital, Leicester LE1 7RH, UK; ^2^Department of Cardiology, Aintree University Hospital, Liverpool, UK; ^3^Department of Cardiology, Poole Hospital NHS Foundation Trust, Poole, UK; ^4^Diabetes Research Centre, University of Leicester and the NIHR Leicester Biomedical Research Centre, Leicester General Hospital, Leicester, UK

**Keywords:** Diastolic Dysfunction, Peak Early Diastolic Strain Rate, Peak Late Diastolic Strain Rate, Feature Tracking

## Abstract

**Purpose:**

To compare left ventricular (LV) peak early diastolic strain rate (PEDSR) and peak late diastolic strain rate (PLDSR) using cardiac MRI feature tracking (FT) across a spectrum of diastolic dysfunction and determine the association between diastolic strain rates and cardiac remodeling.

**Materials and Methods:**

Between October 2008 and December 2022, cardiac MRI and echocardiography were performed in prospectively recruited cohorts with type 2 diabetes mellitus, heart failure with preserved ejection fraction, and severe aortic stenosis, as well as asymptomatic participants without diabetes. Diastolic dysfunction was classified using established echocardiography guidelines. Global circumferential and longitudinal PEDSR and PLDSR were measured at cardiac MRI. Linear regression was performed to identify independent associations between LV diastolic strain rates and remodeling.

**Results:**

A total of 600 participants (mean age, 65.2 years ± 8.4 [SD]; 361 of 600 male participants [60%]) were included. Proportions of participants with normal diastolic function and those with grade 1, indeterminate, and grade 2 or 3 diastolic dysfunction were 92 of 600 (15%), 401 of 600 (67%), 85 of 600 (14%), and 22 of 600 (4%), respectively. Compared with participants who had normal function, PEDSR decreased in those with grade 1 dysfunction (circumferential PEDSR, 0.99 sec^-1^ ± 0.22 vs 0.81 sec^-1^ ± 0.24 [*P* < .001]; longitudinal PEDSR, 0.79 sec^-1^ ± 0.19 vs 0.60 sec^-1^ ± 0.19 [*P* < .001]) and remained low throughout worsening stages of diastolic dysfunction. In contrast, compared with participants who had normal diastolic function, PLDSR increased in those with grade 1 dysfunction (circumferential PLDSR, 0.70 sec^-1^ ± 0.17 vs 0.82 sec^-1^ ± 0.23 [*P* < .001]; longitudinal PLDSR, 0.73 sec^-1^ ± 0.18 vs 0.80 sec^-1^ ± 0.27 [*P* < .001]) and declined progressively with worsening diastolic dysfunction. After multivariable adjustment for risk factors, inverse associations persisted between PEDSR and PLDSR with cardiac remodeling.

**Conclusion:**

A distinctive pattern of cardiac MRI FT early and late diastolic strain rates was observed across the range of diastolic dysfunction.

**Keywords:** Diastolic Dysfunction, Peak Early Diastolic Strain Rate, Peak Late Diastolic Strain Rate, Feature Tracking

*Supplemental material is available for this article.*

© The Author(s) 2025. Published by the Radiological Society of North America under a CC BY 4.0 license.

## Graphical abstract

**Figure:**
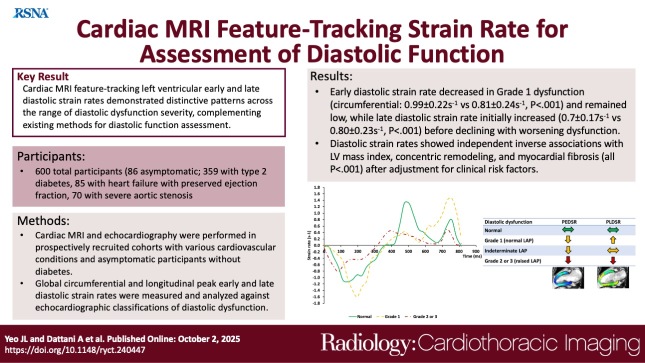

**Summary**

A distinctive pattern of cardiac MRI feature-tracking left ventricular early and late diastolic strain rates was observed across the range of diastolic dysfunction severity.

**Key Points**

■ Peak early diastolic strain rate decreased in participants with grade 1 diastolic dysfunction compared with those who had normal diastolic function (circumferential peak early diastolic strain rate, 0.99 sec^−1^ ± 0.22 vs 0.81 sec^−1 ^± 0.24; *P* < .001) and remained low throughout worsening stages of diastolic dysfunction.
■ Peak late diastolic strain rate increased in participants with grade 1 dysfunction compared with those who had normal diastolic function (circumferential peak late diastolic strain rate, 0.70 sec^−1^ ± 0.17 vs 0.82 sec^−1 ^± 0.23; *P* < .001) and progressively declined thereafter with worsening diastolic dysfunction.
■ Diastolic strain rates were independently associated with markers of adverse cardiac remodeling after multivariable adjustment for clinical risk factors.

## Introduction

Diastolic dysfunction is a hallmark pathophysiologic feature underpinning various cardiovascular conditions, including heart failure with preserved ejection fraction (HFpEF). This condition affects half of the population with heart failure and is increasingly prevalent in parallel with rising obesity and type 2 diabetes mellitus (TD2) (1). Diastolic dysfunction is also a feature of aortic stenosis (AS), which is the most common valvular abnormality in high-income countries (2). The noninvasive modality of choice for assessment of diastolic function is echocardiography, primarily with Doppler measurements of mitral inflow and annular velocities. Echocardiography indexes are supported by a wealth of validation data against invasive measures of left ventricular (LV) end-diastolic pressure and are predictive of poor prognosis (3,4). Although echocardiography is more accessible, its inherent limitations include dependence on acoustic window, poor reproducibility, and operator variability.

Cardiac MRI is the reference standard for evaluation of chamber volumes, in addition to myocardial tissue characterization, perfusion, and viability in an integrated examination, with increasing clinical utility for diagnosis and monitoring of multiple diseases. Assessment of diastolic function with cardiac MRI has traditionally been more challenging but is gaining in popularity (5,6). Several studies have shown the utility of cardiac MRI in assessing diastolic function by using phase-contrast sequence to measure transmitral and myocardial tissue velocities; these studies have found good correlation with echocardiography (7,8). The advent of myocardial deformation imaging has permitted direct assessment of myocardial relaxation. More recently, cardiac MRI feature tracking (FT) allows measurement of strain and strain rates directly from routinely acquired cine imaging. Peak early diastolic strain rate (PEDSR) is a surrogate marker of myocardial stiffness; it has been shown to have good agreement with echocardiography in diagnosing diastolic dysfunction (9) and was associated with adverse outcomes in HFpEF (10). However, peak strain rate can also be quantified in late diastole (PLDSR) coinciding with contraction of the left atrium (LA), but to our knowledge no cardiac MRI studies have investigated this in participants with or at risk for heart failure. Furthermore, the relationship between diastolic strain rates as a functional measure and cardiac MRI measures of cardiac remodeling is unclear.

We hypothesize that cardiac MRI–derived LV early and late diastolic strain rates would decrease with worsening diastolic dysfunction. The aims of this study are to compare early and late diastolic strain rates across a spectrum of diseases and grades of diastolic dysfunction and to determine the association between LV diastolic strain rate indexes and cardiac remodeling.

## Materials and Methods

### Study Participants

This is a pooled cross-sectional analysis of research participants at a single center (Glenfield Hospital, Leicester, United Kingdom) prospectively recruited between October 2008 and December 2022. Four groups were studied: known cardiovascular symptoms or diabetes, T2D (11), HFpEF (12), and AS (13). The eligibility criteria of individual study cohorts are summarized in Table S1. All participants had an estimated glomerular filtration rate greater than 30 mL/min per 1.73 m^2^, had no absolute contraindication to cardiac MRI, and provided written informed consent. Participants who were in atrial fibrillation during the MRI examination were excluded because PLDSR was not measurable. The UK Health Research Authority Research Ethics Committee approved the study (references 17/WM/0192, 12/EM/0222, 19/EM/0032, and 08/H0402/6).

### General Examinations

Demographic characteristics, medical history, and anthropometric measurements were collected. Hypertension and hypercholesterolemia were determined by self-reporting from participants or prescriptions for medication to treat these conditions. A fasting blood sample was collected for biochemical profiling, including kidney function, lipid profile, glycosylated hemoglobin, and natriuretic peptides. Samples were collected on the same day as the imaging procedures and were analyzed in an accredited National Health Service pathology laboratory at the University Hospitals of Leicester.

### Transthoracic Echocardiography

Transthoracic echocardiography was performed using an iE33b system (Philips Medical Systems) or Vivid 7 (GE HealthCare) according to standard reporting guideline (14). Early (E) and late (A) diastolic mitral inflow velocities and early diastolic mitral annular velocities (e′) were assessed with Doppler echocardiography. Maximal LA volume was measured using the biplane Simpson method from the four- and two-chamber long-axis views and was indexed to body surface area.

### Diastolic Dysfunction Classification

Echocardiographic diastolic dysfunction classification was determined using the American Society of Echocardiography guideline as follows (15): *(a)* normal diastolic function: E/A greater than 0.8 to less than 2, septal e′ greater than 7 cm/sec, lateral e′ greater than 10 cm/sec, E/e′ less than 14, and LA volume index (LAVi) less than 34 mL/m^2^; *(b) *grade 1 diastolic dysfunction: E/A 0.8 or less and E of 50 cm/sec or less; *(c) *grade 2: E/A of 0.8 or less and E greater than 50 cm/sec or E/A greater than 0.8 to less than 2 and both E/e′ greater than 14 and LAVi greater than 34 mL/m^2^;* (d)* indeterminate: similar to grade 2 but only positive for either E/ e′ greater than 14 or LAVi greater than 34 mL/m^2^; and *(e)* grade 3: E/A 2 or greater and septal e′ less than 7 cm/sec or lateral e′ less than 10 cm/sec. Tricuspid regurgitation velocity or pulmonary vein flows were not used in our classification because they were not reliably measured in a large proportion of our cohort.

### Cardiac MRI

Cardiac MRI was performed using a standardized protocol on 3-T scanners (Skyra or Vida; Siemens), as described elsewhere (11,12). A proportion of the participants with AS (*n* = 44) were scanned using a 1.5-T scanner (Avanto; Siemens) (13). Steady-state free precession cine images were acquired in the four-, three-, and two-chamber long-axis views and in a short-axis stack to cover the entire LV. Imaging parameters were as follows: *(a)* for 3-T imaging: field of view, 360 × 293 mm; matrix size, 256 × 205; section thickness, 8 mm; flip angle, 80°; repetition time msec/echo time msec, 47.5/1.49; parallel imaging acceleration factor, 3;* (b)* for 1.5-T imaging: field of view, 320 × 260 mm; matrix size, 256 × 204; section thickness, 8 mm; flip angle, 67°; 34.5/1.33; parallel imaging acceleration factor, 2. Images were reconstructed to 30 phases per cardiac cycle. Late gadolinium enhancement (LGE) images were acquired at least 5 minutes after administration of contrast material (0.15 mmol/kg of gadobutrol or gadoterate) for assessment of myocardial infarction and focal myocardial fibrosis. LGE images were acquired in the same positions as long- and short-axes cine images using a phase-sensitive inversion recovery reconstruction sequence.

### Analysis of Cardiac MRI Scans

Cardiac MRI scans were analyzed using cvi42, version 5.10.1 (Circle Cardiovascular Imaging) by one of three trained observers (J.L.Y., A.D., and G.S.G., with 2, 2, and 5 years of cardiac MRI experience at the time of analysis, respectively) blinded to participant demographic and clinical details. LV mass and volumes were measured using the short-axis stack. Papillary muscles were excluded from the quantification of LV mass. LV mass to end-diastolic volume ratio (LVM/V) was calculated as a marker of LV concentric remodeling. The biplane method from four- and two-chamber long-axis views, excluding the appendage and pulmonary veins, was used to measure maximal LA volume.

LV strain parameters, including peak global circumferential systolic strain, global longitudinal systolic strain, PEDSR, and PLDSR, were obtained by automated tracking of the myocardial voxel points. Semiautomated contouring of the endocardial and epicardial borders of the four-, three-, and two-chamber long-axis cines were performed for longitudinal strain and short-axis stack cines for circumferential strain. The end-diastolic phase was the starting point of strain tracking. Visual inspection across all phases was conducted to ensure accurate tissue tracking and manual adjustments were performed if necessary. PEDSR was defined as the first peak of the strain rate curve during diastole corresponding to early passive LV filling. PLDSR was defined as the second peak of the strain rate curve, corresponding to LV filling during atrial contraction. Strain and strain rates are presented as absolute values, where lower values indicate lower myocardial deformation, to avoid confusion with negative values, which represent myocardial shortening (16). Radial strain was not quantified in this study because of the poor reproducibility, as reported elsewhere (17). LGE images were assessed qualitatively for ischemic scarring (subendocardial or transmural LGE) and nonischemic fibrosis (intramural or subepicardial LGE). Pulmonary capillary wedge pressure (PCWP) was estimated using a recently validated formula: 6.1352 + (0.07204 × left atrial volume) + (0.02256 × LVM) (18).

### Statistical Analysis

Continuous data are presented as means ± sds or medians (25th–75th percentile ranges). Categorical data were presented as absolute numbers with percentages. Normality for continuous data was assessed using histograms and Q-Q plots. Comparison of imaging parameters between participants with different grades of diastolic dysfunction severity was performed using one-way analysis of variance with a Bonferroni post hoc test if variances were equal between groups or a Games–Howell post hoc test if variances were unequal. Linear regression analysis was performed in participants with diastolic dysfunction to identify univariable and multivariable associations between circumferential and longitudinal PEDSR and PLDSR with cardiac MRI measures of remodeling (LVM index, LVM/V, LA volume index, nonischemic LGE) and estimated PCWP. Each cardiac MRI measure of remodeling was tested separately in a model adjusting for covariates including age, sex, hypertension, heart rate, and presence of myocardial infarction at LGE imaging.

Interobserver variability for three observers and intraobserver variability were assessed in 10 randomly selected participants from the cohort. Comparisons were made using paired-sample *t* tests for intraobserver, repeated-measures analysis of variance for interobserver, and intraclass correlation coefficient in a two-way mixed-effects model. Intraclass correlation coefficient was scored as follows: excellent, greater than 0.90; good, >0.75–0.90; moderate, 0.50–0.75; poor, less than 0.50 (19). Statistical analyses were performed using Statistical Package for Social Services, version 28.0 (SPSS). A *P* value less than .05 was considered to indicate statistical significance.

## Results

### Clinical Characteristics

The study flowchart and reasons for exclusions are presented in Figure 1. Of the 615 participants with available cardiac MRI data, 15 participants (2%) did not have sufficient echocardiography data to reliably determine diastolic function and were excluded (Fig 2). Final analysis included 600 individuals: 86 asymptomatic participants without T2D, 359 with asymptomatic T2D, 85 with HFpEF, and 70 with AS. Baseline clinical characteristics are summarized in Table 1. Mean age was 65.2 years ± 8.4, 361 of 600 (60%) were male, and mean body mass index (calculated as weight in kilograms divided by height in meters squared) was 30.0 ± 5.9. There was a substantial burden of hypertension in participants with HFpEF (76 of 85 [89%]).

**Figure 1:**
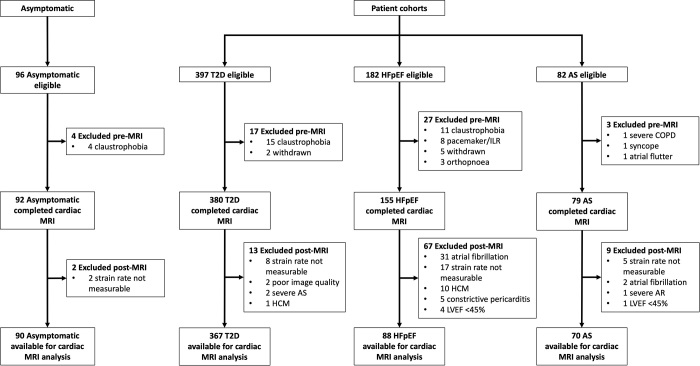
Flowchart displays how participants, recruited between October 2008 and December 2022, were selected for analysis and details of exclusions. AR = aortic regurgitation, AS = aortic stenosis, COPD = chronic obstructive pulmonary disease, HCM = hypertrophic cardiomyopathy, HFpEF = heart failure with preserved ejection fraction, ILR = implantable loop recorder, LVEF = left ventricular ejection fraction, T2D = type 2 diabetes mellitus.

**Figure 2:**
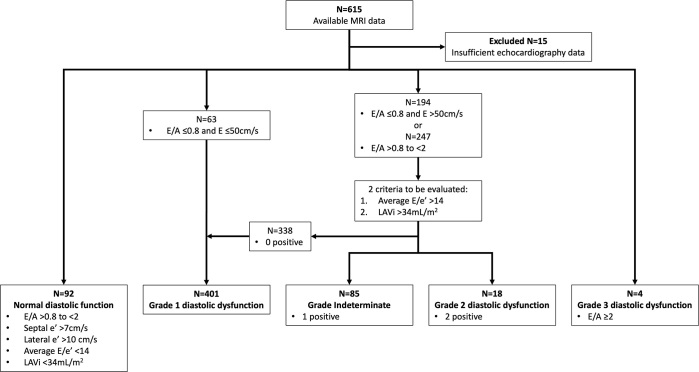
Flowchart demonstrates the algorithm using established echocardiography guidelines to determine diastolic dysfunction grades. E/A = early-to-late diastolic mitral inflow velocity ratio, E/e′ = early diastolic mitral inflow velocity to mitral annular velocity ratio, e′ = early diastolic mitral annular velocity, LAVi = left atrial volume index.

**Table 1:** Baseline Characteristics of Overall and Individual Disease Cohorts

Characteristic	Overall (*n* = 600)	Asymptomatic (*n* = 86)	T2D (*n* = 359)	HfpEF (*n* = 85)	AS (*n* = 70)
Demographics and medical history					
Age (y)	65.2 ± 8.4	67.1 ± 9.1	63.2 ± 6.8	70.7 ± 9.5	66.6 ± 9.5
Female	239 (40)	37 (43)	138 (38)	46 (54)	18 (26)
Male	361 (60)	49 (57)	221 (62)	39 (46)	52 (74)
Ethnicity					
Black and mixed races	17 (3)	1 (1)	12 (3)	3 (4)	1 (1)
South Asian	110 (18)	14 (16)	76 (21)	15 (18)	5 (7)
White	473 (79)	71 (83)	271 (76)	67 (79)	64 (91)
Smoking status					
Never smoked	318 (54)	52 (61)	203 (57)	41 (48)	22 (37)
Formerly smoked	239 (41)	34 (40)	134 (37)	37 (44)	34 (57)
Currently smokes	33 (6)	0	22 (6)	7 (8)	4 (7)
BMI*	30.0 ± 5.9	26.0 ± 3.6	30.1 ± 5.5	34.2 ± 7.2	29.5 ± 5.1
Hypertension	353 (59)	29 (34)	202 (56)	76 (89)	46 (66)
Hypercholesterolemia	337 (56)	24 (28)	246 (69)	43 (51)	24 (34)
TD2	436 (71)	0	359 (100)	48 (57)	19 (27)
Paroxysmal AF	17 (3)	0	0	17 (20)	0
Systolic BP (mm Hg)	138.6 ± 20.5	145.6 ± 22.8	135.4 ± 16.8	147.0 ± 26.9	135.2 ± 20.8
Diastolic BP (mm Hg)	79.2 ± 10.6	82.1 ± 10.4	80.8 ± 9.4	72.4 ± 12.8	75.8 ± 9.9
Heart rate (beats/min)	73.4 ± 13.2	66.1 ± 10.0	76.5 ± 12.6	69.5 ± 14.0	66.5 ± 13.4
Medication					
ACE inhibitor/ARB	287 (48)	15 (17)	167 (47)	79 (93)	26 (38)
β-blocker	115 (19)	2 (2)	25 (7)	56 (66)	32 (46)
Calcium channel blocker	137 (26)	14 (16)	96 (27)	27 (32)	0
Statin	358 (65)	9 (21)	248 (70)	54 (64)	47 (68)
Insulin	72 (14)	0	54 (15)	17 (20)	1 (4)
Biochemistry					
Estimated GFR (mL/min per 1.73 m^2^)	83.2 ± 17.0	89.3 ± 11.8	87.8 ± 13.2	66.3 ± 22.2	72.8 ± 14.9
Hemoglobin A_1c_ (%)	7.0 ± 1.3	5.5 ± 0.3	7.4 ± 1.3	6.7 ± 1.2	6.3 ± 0.6
Total cholesterol (mmol/L)	4.5 ± 1.2	5.5 ± 1.1	4.3 ± 1.1	4.7 ± 1.5	4.4 ± 1.0
NT-proBNP (ng/L)	56 (24–117)	69 (39–103)	45 (0–76)	221 (90–652)	1850 (601–4341)
Echocardiographic diastolic dysfunction grade					
Normal	92 (15)	18 (21)	73 (20)	0	1 (1)
Grade 1	401 (67)	56 (65)	261 (73)	43 (51)	41 (59)
Indeterminate	85 (14)	12 (14)	24 (7)	29 (34)	20 (29)
Grade 2	18 (3)	0	0	11 (13)	7 (10)
Grade 3	4 (1)	0	1 (0)	2 (2)	1 (1)

Note.—Values are expressed as means ± sds, numbers of participants with percentages in parentheses, or medians with IQRs in parentheses. ACE = angiotensin-converting enzyme, AF = atrial fibrillation, ARB = angiotensin receptor blocker, BMI = body mass index, BP = blood pressure, GFR = glomerular filtration rate, NT-proBNP = N-terminal prohormone brain natriuretic peptide. To convert cholesterol values to milligrams per deciliter, divide by 0.0259. To convert NT-proBNP values to picograms per milliter, multiply by 1.

* BMI was calculated as weight in kilograms divided by height in meters squared.

### Echocardiography and Cardiac MRI Data

Of the 600 participants, 92 (15%) had normal diastolic function, 401 (67%) had grade 1 dysfunction, 85 (14%) had indeterminate dysfunction, and 22 (4%) had grade 2 or 3 dysfunction (Table 1). No participants with HFpEF and only one with AS had normal diastolic function. No asymptomatic participants with T2D had grade 2 diastolic dysfunction. One participant with T2D had grade 3 dysfunction, as did two participants with HFpEF and one with AS. Echocardiography and cardiac MRI data by participant group are shown in Table S2; echocardiographic diastolic function showed a worsening trend from asymptomatic participants to those with T2D and then a further decrease in those with HFpEF or AS. LV concentric remodeling was greatest in those with AS, followed by participants with T2D and HFpEF, with the increased LVM/V primarily attributed to lower LVEDV in the former compared with asymptomatic participants. Global systolic strain tended to reduce from asymptomatic participants to T2D to HFpEF/AS as did diastolic strain rates.

Echocardiography and cardiac MRI data stratified by diastolic dysfunction severity are presented in Table 2. As diastolic dysfunction worsened, there was a pattern of progressively higher LVM and LVEDV index. LV remodeling, represented by LVM/V, was higher with grade 1 and indeterminate diastolic dysfunction compared with normal. Systolic function was also gradually impaired with worsening diastolic dysfunction, as shown by lower global circumferential systolic strain and global longitudinal systolic strain. There was a progressive increase in nonischemic LGE with increasing diastolic dysfunction. Estimated PCWP derived from formula also increased with diastolic dysfunction (Table S3).

**Table 2:** Demographic and Imaging Characteristics according to Diastolic Dysfunction Severity

Parameter	Diastolic Dysfunction Severity				
	Normal (*n* = 92)	Grade 1 (*n* = 401)	Indeterminate (*n* = 85)	Grade 2 or 3 (*n* = 22)	*P* Value
Age (y)	59.9 ± 7.7	65.0 ± 7.8*	70.4 ± 7.2*^†^	71.9 ± 9.6*^†^	<.001
Female	25 (27)	162 (40)	44 (52)	8 (36)	.020
Male	67 (73)	239 (60)	41 (48)	14 (64)	.010
Echocardiography					
E (cm/sec)	77.9 ± 12.6	64.8 ± 14.4*	83.8 ± 18.8^†^	103.3 ± 29.9*^†^^‡^	<.001
A (cm/sec)	72.7 ± 13.7	81.9 ± 16.3*	93.9 ± 22.4*^†^	97.0 ± 40.2*	<.001
E/A	1.09 ± 0.20	0.81 ± 0.18*	0.92 ± 0.24*^†^	1.28 ± 0.69^†^	<.001
Septal e′ (cm/sec)	8.6 ± 1.1	6.4 ± 1.5*	5.8 ± 2.8*	4.6 ± 1.5*^†^	<.001
Lateral e′ (cm/sec)	12.1 ± 1.7	8.5 ± 2.0*	7.5 ± 2.6*^†^	6.9 ± 2.4*	<.001
Mean E/e′	7.9 ± 1.4	9.3 ± 2.0*	14.2 ± 4.4*^†^	20.0 ± 6.0*^†^^‡^	<.001
LA volume indexed to BSA (mL/m^2^)	22.7 ± 5.1	23.2 ± 6.2	34.7 ± 13.1*^†^	45.1 ± 12.4*^†^^‡^	<.001
Cardiac MRI					
LVM indexed to height (g/m)	64.8 ± 10.1	71.4 ± 17.9*	79.9 ± 23.5*^†^	94.2 ± 40.0*	<.001
LVEDV indexed to height (mL/m)	77.3 ± 14.3	79.0 ± 18.7	87.6 ± 19.1*^†^	94.8 ± 14.5*^†^	<.001
LVM/V (g/mL)	0.85 ± 0.11	0.92 ± 0.17*	0.92 ± 0.24*	0.99 ± 0.37	.002
GCS (%)	19.5 ± 2.1	19.1 ± 2.7	19.0 ± 3.1	17.8 ± 3.0	.056
GLS (%)	16.9 ± 2.2	15.8 ± 2.5*	15.2 ± 2.9*	13.8 ± 3.9*	<.001
Ischemic LGE	1 (1)	16 (4)	9 (11)	2 (9)	.013
Nonischemic LGE	8 (9)	98 (25)	32 (38)	12 (55)	<.001
Cardiac MRI diastolic strain rates					
Circumferential PEDSR (sec^−1^)	0.99 ± 0.22	0.81 ± 0.24*	0.76 ± 0.25*	0.65 ± 0.19*^†^	<.001
Circumferential PLDSR (sec^−1^)	0.70 ± 0.17	0.82 ± 0.23*	0.70 ± 0.23^†^	0.49 ± 0.18*^†^^‡^	<.001
Circumferential PEDSR/PLDSR	1.50 ± 0.51	1.08 ± 0.44*	1.20 ± 0.55*	1.67 ± 1.34	<.001
Longitudinal PEDSR (sec^−1^)	0.79 ± 0.19	0.60 ± 0.19*	0.59 ± 0.20*	0.50 ± 0.19*	<.001
Longitudinal PLDSR (sec^−1^)	0.73 ± 0.18	0.80 ± 0.27*	0.67 ± 0.23^†^	0.48 ± 0.16*^†^^‡^	<.001
Longitudinal PEDSR/PLDSR	1.14 ± 0.36	0.80 ± 0.31*	0.95 ± 0.39*^†^	1.26 ± 0.97	<.001

Note.—Values are expressed as means ± sds or numbers of participants, with percentages in parentheses. A = late diastolic mitral inflow velocity, BSA = body surface area, E = early diastolic mitral inflow velocity, e′ = mitral annular velocity, GCS = global circumferential systolic strain, GLS = global longitudinal systolic strain, LA = left atrium, LGE = late gadolinium enhancement, LVEDV = left ventricular end-diastolic volume, LVM = left ventricular mass, LVM/V = left ventricular mass to end-diastolic volume ratio, MI = myocardial infarction, PEDSR = peak early diastolic strain rate, PLDSR = peak late diastolic strain rate.

* *P* < .05 versus normal.

^†^ *P* < .05 versus grade 1.

^‡^ *P* < .05 versus indeterminate.

### Early Diastolic Strain Rates

Example images of strain assessment are illustrated in Figure 3. Strain rate data by echocardiographic diastolic grading are illustrated in Figure 4. Compared with normal diastolic function, both circumferential and longitudinal PEDSR were lower in participants with diastolic dysfunction. Longitudinal PEDSR was similar among the different grades of diastolic dysfunction, with a trend toward reducing circumferential PEDSR with increasing diastolic dysfunction.

**Figure 3:**
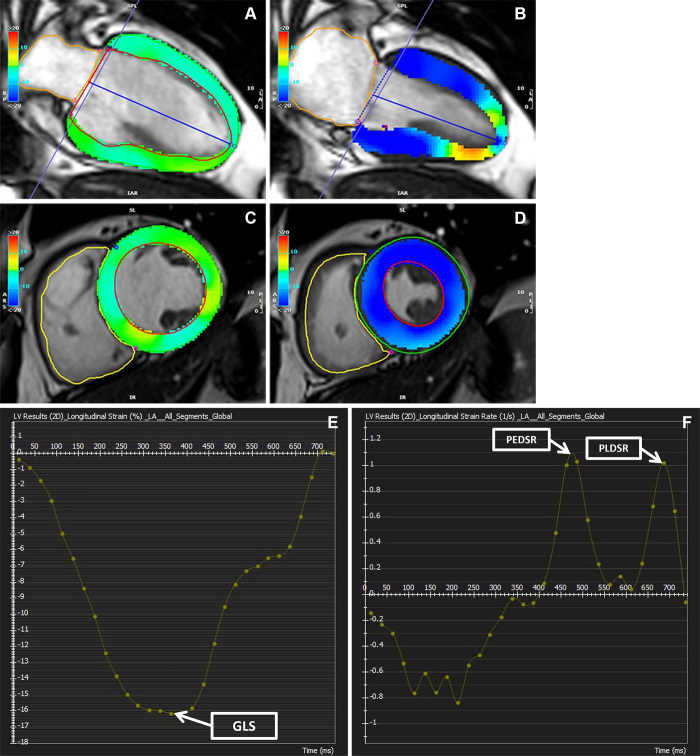
Example cardiac MR images of myocardial deformation analysis. **(A, B)** Longitudinal strain analysis in the end-diastolic and end-systolic phases of the two-chamber view. **(C, D)** Circumferential strain analysis in the end-diastolic and end-systolic phases of the midventricular short axis section. Strain is color-coded; darker blue indicates better systolic strain. **(E)** Global longitudinal strain and **(F)** strain rate curves across all phases of cardiac cycle. GLS = peak global longitudinal systolic strain, LA = left atrium, LV = left ventricle, PEDSR = peak early diastolic strain rate, PLDSR = peak late diastolic strain rate, 2D = two-dimensional.

**Figure 4:**
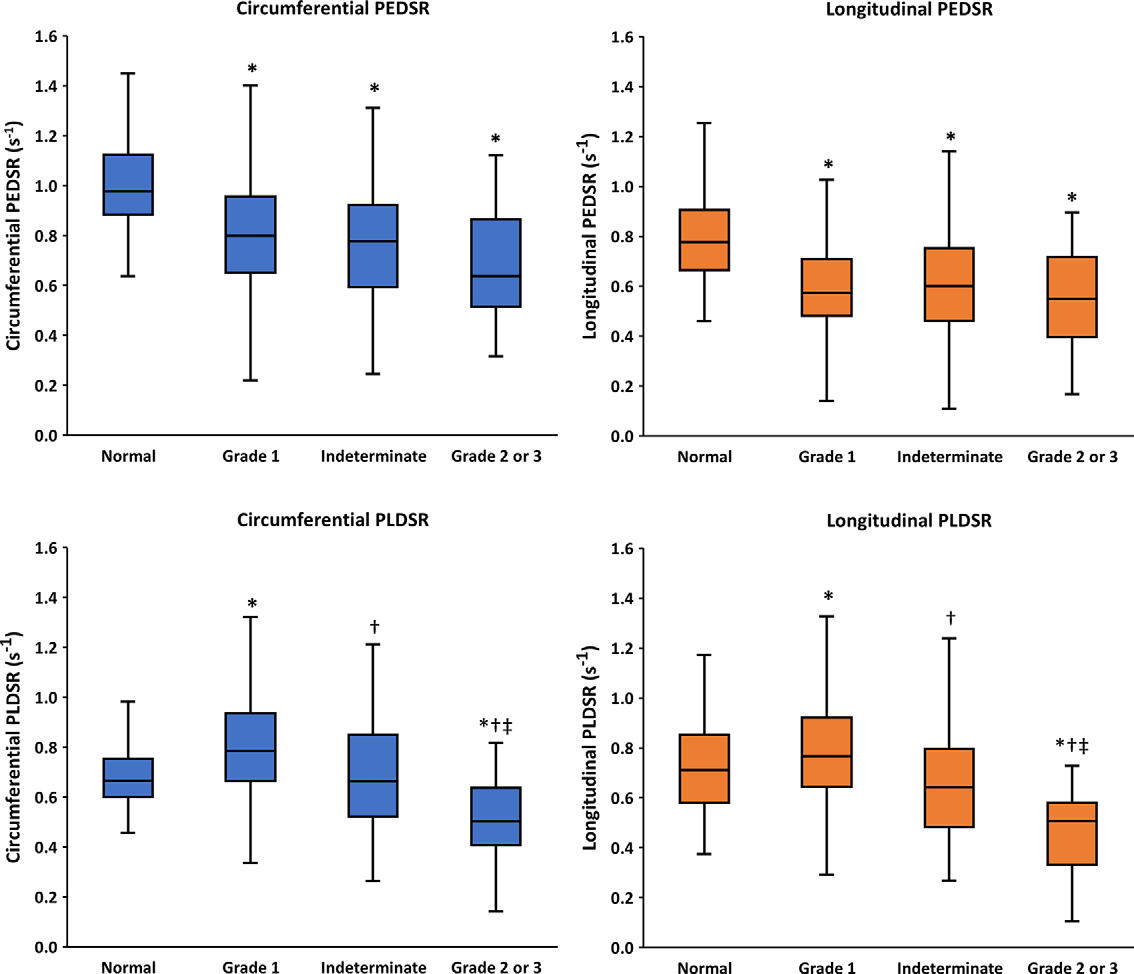
Boxplots demonstrate circumferential and longitudinal peak early diastolic strain rate (PEDSR) and peak late diastolic strain rate (PLDSR) stratified by echocardiography-determined diastolic dysfunction grading. *P < .05 versus normal, †*P* < .05 versus grade 1, ‡*P* < .05 versus indeterminate. Horizontal lines of the boxplots are the 25th, median, and 75th percentiles. Whiskers are the minimum and maximum values.

### Late Diastolic Strain Rates

Circumferential and longitudinal PLDSR were higher in participants with grade 1 dysfunction than those with normal diastolic function and were progressively lower from indeterminate dysfunction to grade 2 or 3 diastolic dysfunction. Representative strain rate curves of participants with normal and diastolic dysfunction are displayed in Figure 5.

**Figure 5:**
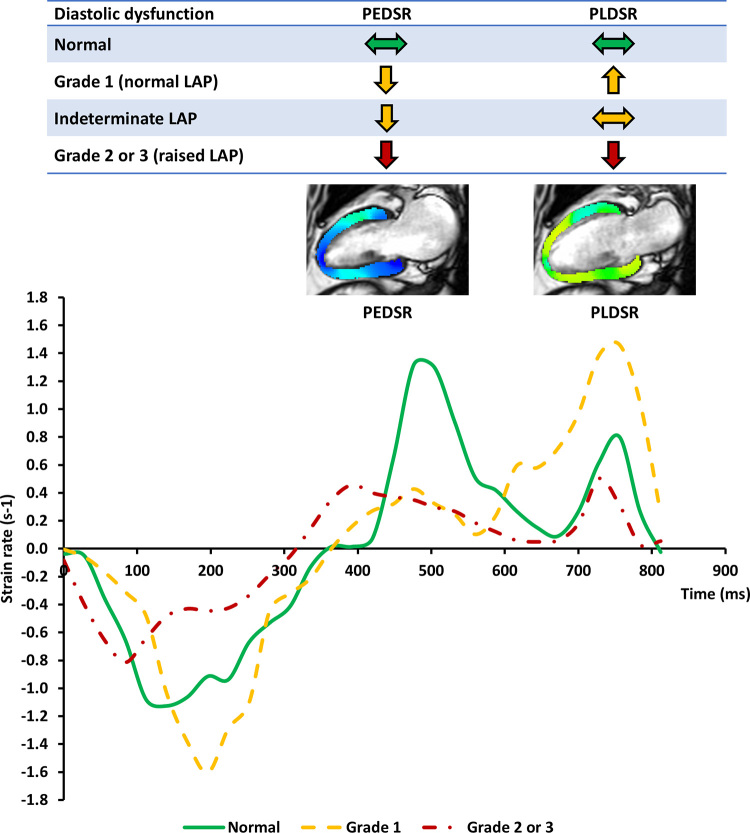
Representative strain rate curves across the spectrum of diastolic dysfunction. As diastolic dysfunction progresses, peak early diastolic strain rate (PEDSR) decreases and remains low, whereas peak late diastolic strain rate (PLDSR) initially increases in early dysfunction (grade 1) and then progressively declines. LAP = left atrial pressure.

### Early-to-Late Diastolic Strain Rate Ratios

Both circumferential and longitudinal PEDSR/PLDSR were lower in participants with grade 1 dysfunction but increased with worsening diastolic dysfunction.

### Association between Strain Rates and Cardiac Remodeling

Univariable and multivariable association between PEDSR and PLDSR with cardiac remodeling are displayed in Table 3 and 4. There were inverse univariable correlations between PEDSR with LV remodeling and PLDSR with LA remodeling. PEDSR and PLDSR were also inversely associated with nonischemic LGE. These inverse relationships remained significant after multivariable adjustments. There were also inverse associations between PEDSR and PLDSR with cardiac MRI–estimated PCWP (Tables S4, S5).

**Table 3:** Associations between Peak Early Diastolic Strain Rate with Left Ventricular Remodeling in Participants with Diastolic Dysfunction

Variable	Univariable Analysis		Multivariable Analysis					
			Model 1		Model 2		Model 3	
	β	*P* Value	β	*P* Value	β	*P* Value	β	*P* Value
Circumferential PEDSR								
Age	-0.25	<.001	−0.29	<.001	−0.23	<.001	−0.24	<.001
Sex (male)	-0.30	<.001	-0.18	<.001	-0.25	<.001	-0.27	<.001
Hypertension	-0.12	.010	0.01	.853	-0.02	.689	-0.03	.537
Heart rate	0.13	.005	-0.05	.255	0.09	.057	0.00	.962
MI (on LGE)	-0.10	.027	-0.06	.157	-0.07	.093	-0.06	.137
LVM/height	-0.37	<.001	-0.33	<.001				
LVM/V	-0.25	<.001			-0.24	<.001		
Nonischemic LGE	-0.23	<.001					-0.16	<.001
*R^2^*			0.24		0.21		0.19	
Longitudinal PEDSR								
Age	-0.20	<.001	-0.24	<.001	-0.19	<.001	-0.20	<.001
Sex (male)	-0.30	<.001	-0.20	<.001	-0.25	<.001	-0.27	<.001
Hypertension	-0.03	.568	0.08	.083	0.05	.211	0.05	.302
Heart rate	0.18	<.001	0.03	.551	0.16	<.001	0.07	.134
MI (on LGE)	-0.01	.901	0.03	.527	0.02	.732	0.01	.882
LVM/height	-0.34	<.001	-0.29	<.001				
LVM/V	-0.23	<.001			-0.23	<.001		
Nonischemic LGE	-0.22	<.001					-0.14	.002
*R*^2^			0.21		0.19		0.16	

Note.—β is standardized coefficient. LGE = late gadolinium enhancement, LVM = left ventricular mass, LVM/V = left ventricular mass to end-diastolic volume ratio, MI = myocardial infarction, PEDSR = peak early diastolic strain rate.

**Table 4:** Associations between Peak Late Diastolic Strain Rate with Cardiac Remodeling in Participants with Diastolic Dysfunction

Variable	Univariable Analysis		Multivariable Analysis							
			Model 1		Model 2		Model 3		Model 4	
	β	*P* Value	β	*P* Value	β	*P* Value	β	*P* Value	β	*P* Value
Circumferential PLDSR										
Age	0.07	.151	0.17	<.001	0.23	<.001	0.23	<.001	0.23	<.001
Sex (male)	-0.09	.047	0.13	.002	0.02	.731	-0.02	.637	0.01	.780
Hypertension	-0.04	.378	-0.01	.753	-0.05	.205	-0.02	.677	-0.06	.176
Heart rate	0.45	<.001	0.43	<.001	0.54	<.001	0.39	<.001	0.50	<.001
MI (on LGE)	-0.12	.007	-0.09	.017	-0.10	.016	-0.08	.042	-0.09	.033
LVM/height	-0.44	<.001	-0.37	<.001						
LVM/V	0.02	.643			-0.10	.018				
LA volume/height	-0.41	<.001					-0.30	<.001		
Nonischemic LGE	-0.18	.004							-0.10	.022
*R*^2^			.37		0.27		.33		0.27	
Longitudinal PLDSR										
Age	-0.06	.166	0.02	.600	0.07	.118	0.07	.130	0.06	.154
Sex (male)	0.17	<.001	0.00	.991	-0.08	.075	-0.12	.006	-0.07	.088
Hypertension	-0.09	.054	-0.03	.435	-0.06	.150	-0.04	.382	-0.06	.136
Heart rate	0.40	<.001	0.33	<.001	0.44	<.001	0.30	<.001	0.37	<.001
MI (on LGE)	-0.12	.008	-0.08	.057	-0.09	.043	-0.07	.099	-0.09	.039
LVM/height	-0.39	<.001	-0.30	<.001						
LVM/V	-0.05	.313			-0.13	.004				
LA volume/height	-0.35	<.001					-0.23	<.001		
Nonischemic LGE	-0.24	<.001							-0.16	<.001
*R^2^*			0.26		0.20		0.23		0.21	

Note.—β is standardized coefficient. LA = left atrium, LGE = late gadolinium enhancement, LVM = left ventricular mass, LVM/V = left ventricular mass to end-diastolic volume ratio, MI = myocardial infarction, PLDSR = peak late diastolic strain rate.

### Inter- and Intraobserver Variability

The inter- and intraobserver variability of cardiac MRI diastolic strain parameters are presented in Tables S6 and S7. Interobserver reproducibility was excellent for circumferential PEDSR and moderate for the other strain rate measures. Intraobserver reproducibility was good to excellent for all strain rate measures.

## Discussion

This is the first study to our knowledge to characterize the pattern of myocardial lusitropy by cardiac MRI FT early and late diastolic strain rates across the spectrum of diastolic dysfunction severity in several disease states. We found that PEDSR decreased in participants who had grade 1 dysfunction compared with those who had normal function and remained low throughout worsening stages of diastolic dysfunction. In contrast, PLDSR increased in those who had mild diastolic dysfunction (grade 1) compared with those who had normal function, and it progressively declined thereafter. We also demonstrated that in participants with diastolic dysfunction, PEDSR and PLDSR were independently associated with cardiac remodeling. These findings provide initial evidence supporting the use of combined PEDSR and PLDSR to identify and distinguish between stages of diastolic dysfunction using cardiac MRI FT.

Myocardial deformation imaging plays an increasingly important role in the assessment of diastolic function. Several smaller studies have shown that cardiac MRI FT PEDSR was lower in asymptomatic participants with T2D than in healthy controls (20–22), suggesting that strain rate is a sensitive measure to detect subclinical dysfunction in those at risk for developing heart failure. Furthermore, Mahmod et al (23) reported that among people with HFpEF (*n* = 27; mean age, 72 years ± 7; nine of 27 [33%] male), cardiac MRI–derived circumferential PEDSR was lower than that in controls, was directly associated with myocardial steatosis, and was inversely associated with peak oxygen consumption during exercise. Of note, in another study (10) among participants with HFpEF (*n* = 186; mean age, 59 years ± 12; 109 of 186 [59%] male), a lower longitudinal PEDSR was associated with heart failure hospitalization or all-cause death. Although PEDSR has been widely reported, few studies have examined strain rate in late diastole corresponding to atrial contraction. Atrial booster pump function in late diastole, which corresponds to the same cardiac cycle phase as LV PLDSR, is an important contributor to LV preload in the presence of early LV dysfunction as atrial conduit function cannot provide compensatory ventricular filling (24).

The pattern of diastolic mitral annular velocity in patients with diastolic dysfunction was well described in the early literature (25). Our results agree with the phenomenon observed with echocardiography mitral annulus tissue Doppler velocities, wherein e′ decreases and a′ (late diastolic mitral annular velocity) increases in mild diastolic dysfunction, whereas in severe diastolic dysfunction, both e′ and a′ are lower compared with normal (26). LV filling is predominantly constituted by early mitral inflow in normal diastolic function. With impaired myocardial relaxation but normal LA pressure (grade 1), early transmitral pressure gradient is reduced with increased passive LV filling time, thus increasing LA volume and leading to enhanced LA contractility and a higher PLDSR. As LV pressure and LA afterload increase in grade 2 or 3 dysfunction, mitral A velocity decreases along with a lower PLDSR.

Similar alterations in early and late diastolic strain rates in diastolic dysfunction have been detected by myocardial deformation assessment at echocardiography. A study by Zhu et al (27) assessed LV global longitudinal strain rates during early and late diastole using two-dimensional speckle-tracking echocardiography in young patients undergoing peritoneal dialysis (*n* = 30; mean age, 45 years). They found that early diastolic strain rate was lower and late diastolic strain rate was higher in participants with dialysis compared with age- and sex-matched controls and that early diastolic strain rate was independently associated with LV posterior wall thickness.

In accordance with our results, a previous study using a different cardiac MRI method showed an increase in LV filling rate during late diastole in participants with diastolic dysfunction (28). With use of three-dimensional reconstruction and volume tracking of the mitral annulus, a higher mitral annular sweep rate (representing LV filling) was seen in late diastole among those with diastolic dysfunction compared with those who had normal diastolic function. However, these results were not stratified by severity of dysfunction, and most of the participants in that study may have had mild diastolic dysfunction.

Our data further expand the knowledge in this field by showing that PLDSR is similar to normal in participants with indeterminate LA pressure and lower in those with raised LA pressure (grade 2 or 3), thus providing a discerning attribute with which to classify diastolic dysfunction severity. In participants with low PEDSR, a high PLDSR likely indicated grade 1 diastolic dysfunction; presence of both low PEDSR and PLDSR indicated higher degrees of diastolic dysfunction (Fig 5), although exact cutoffs for age, sex, and ethnicity remain to be determined.

We have also shown independent inverse associations between PEDSR and PLDSR with adverse cardiac remodeling related to diastolic dysfunction. PEDSR and PLDSR were inversely associated with LV mass index, concentric remodeling (LVM/V), estimated LA pressure (cardiac MRI PCWP), and myocardial fibrosis (nonischemic LGE). In addition, PLDSR was inversely associated with LA volume index. These measures of LV and LA remodeling have been previously shown to be key predictors of adverse cardiovascular outcomes (29–31).

Several groups have attempted to mirror the echocardiographic techniques by acquiring transmitral flow and annular velocities with cardiac MRI phase-contrast imaging (7–9). None of these studies focused on the pattern of early and late myocardial diastolic strain rates. These studies are further limited by relatively small numbers and confounded by variable duration between cardiac MRI and echocardiography. Cardiac MRI FT is advantageous over phase-contrast imaging of transmitral flow velocities for the assessment of diastolic function because it can be measured on cine images routinely acquired for volumetric assessment, obviating the need to perform extra scanning sequences. In addition, cardiac MRI FT can provide global and segmental strain rates, which is especially useful in conditions associated with patchy myocardial fibrosis. Moreover, novel machine learning algorithms permit rapid and accurate fully automated segmentation of myocardial borders, thereby substantially improving workflow efficiency (32). Although imperfect, echocardiography remains the clinical standard for noninvasive diastolic function assessment. The growing use of cardiac MRI across a breadth of guideline-recommended (class I) indications, however, makes integration of diastology highly desirable. Using cardiac MRI FT for this purpose could obviate the need for additional testing with echocardiography purely for diastolic function; improve diagnostic capabilities of cardiac MRI (eg, in HFpEF); and expand indications for cardiac MRI, particularly in patients with poor acoustic windows.

Another promising development is the emergence of “direct” atrial strain measures, which have already been incorporated into echocardiography guidelines for diastolic function assessment (33). Although atrial strain may have potential in the future, this technique is limited by poor reproducibility (34). The reproducibility data if “indirect” PLDSR measure of atrial booster pump function are more reassuring, probably because the LV myocardium is easier to track than the LA.

Key strengths of this study include the large prospectively recruited and deeply phenotyped participants across multiple disease states typically associated with diastolic dysfunction. We performed same-day comprehensive echocardiography and state-of-the-art cardiac MRI. We excluded participants with overt systolic impairment, which is an important confounder in assessment of diastolic function. Of note, our aim was not to compare diastolic strain rates across disease states but rather across the progression of diastolic dysfunction.

Our study had several limitations. First, a technical limitation is the lack of measurable PLDSR in the presence of atrial fibrillation, which is prevalent among those with LA dilatation and severe diastolic dysfunction. Second, because this is a cross-sectional study, we do not have outcome data. Further longitudinal studies are needed to determine the association of cardiac MRI FT–derived PEDSR and PLDSR with adverse outcomes. Third, although the number of participants with grade 2 or 3 dysfunction in our study was based on American Society of Echocardiography criteria, it was small and would therefore limit the statistical power of the study; as a result, these participants were grouped in our analysis. Fourth, we have estimated PCWP according to an equation generated in symptomatic patients undergoing invasive investigation, but this has not been validated in asymptomatic participants (18). Further studies are needed to directly validate these cardiac MRI indexes of diastolic function with invasive hemodynamic measurements. Fifth, we used two different cardiac MRI field strengths, wherein a proportion of participants were scanned at 1.5 T (*n* = 44 participants with AS). Sixth, parametric mapping was available only in a subset of our participants; the presence of multiple confounders of these measures, including several disease states across different MRI field strengths (1.5 T and 3 T), introduces more heterogeneity. We have therefore not included T1 mapping data in the current analysis.

In conclusion, early and late diastolic strain rates, measured using cardiac MRI FT, exhibited a distinctive pattern across the range of diastolic dysfunction severity and are independently associated with adverse cardiac remodeling. This technique is incremental to existing methods for the assessment of diastolic function at cardiac MRI. Further studies are required to establish normal reference ranges of diastolic strain rates in healthy population and determine associations with cardiovascular outcomes.
